# Proteomic association with age-dependent sex differences in Wisconsin Card Sorting Test performance in healthy Thai subjects

**DOI:** 10.1038/s41598-023-46750-4

**Published:** 2023-11-19

**Authors:** Chen Chen, Bupachad Khanthiyong, Benjamard Thaweetee-Sukjai, Sawanya Charoenlappanit, Sittiruk Roytrakul, Samur Thanoi, Gavin P. Reynolds, Sutisa Nudmamud-Thanoi

**Affiliations:** 1 Medical Science Graduate Program, Faculty of Medical Science, Naresuan University, Phitsanulok, Thailand; 2 Faculty of Medicine, Bangkokthonburi University, Bangkok, Thailand; 3 School of Medicine, Mae Fah Luang University, Chiang Rai, Thailand; 4 National Centre for Genetic Engineering and Biotechnology, National Science and Technology Development Agency, Pathum Thani, Thailand; 5 School of Medical Sciences, University of Phayao, Phayao, Thailand; 6 Biomolecular Sciences Research Centre, Sheffield Hallam University, Sheffield, UK; 7 Centre of Excellence in Medical Biotechnology, Faculty of Medical Science, Naresuan University, Phitsanulok, Thailand; 8 Department of Anatomy, Faculty of Medical Science, Naresuan University, Phitsanulok, Thailand

**Keywords:** Neuroscience, Psychology

## Abstract

Sex differences in cognitive function exist, but they are not stable and undergo dynamic change during the lifespan. However, our understanding of how sex-related neural information transmission evolves with age is still in its infancy. This study utilized the Wisconsin Card Sorting Test (WCST) and the label-free proteomics method with bioinformatic analysis to investigate the molecular mechanisms underlying age-related sex differences in cognitive performance in 199 healthy Thai subjects (aged 20–70 years), as well as explore the sex-dependent protein complexes for predicting cognitive aging. The results showed that males outperformed females in two of the five WCST sub-scores: %Corrects and %Errors. Sex differences in these scores were related to aging, becoming noticeable in those over 60. At the molecular level, differently expressed individual proteins and protein complexes between both sexes are associated with the potential N-methyl-D-aspartate type glutamate receptor (NMDAR)-mediated excitotoxicity, with the NMDAR complex being enriched exclusively in elderly female samples. These findings provided a preliminary indication that healthy Thai females might be more susceptible to such neurotoxicity, as evidenced by their cognitive performance. NMDAR protein complex enrichment in serum could be proposed as a potential indication for predicting cognitive aging in healthy Thai females.

## Introduction

Cognitive function refers to the higher-order intellectual processes that gather and process information in the human brain. It contains multiple mental abilities, including language, memory, attention, problem-solving, and decision-making, to name a few^1^. At the molecular level, intact cognitive function is dependent on the precise exchange of information between neurons, which is initiated by the activation of excitatory neurotransmitter pathways^2,3^, primarily the glutamatergic pathway, because glutamate (Glu) is the major excitatory neurotransmitter in the central nervous system (CNS). However, its excessive activation would result in excitotoxicity that causes acute neuronal cell damage^4,5^, and Glu is considered a potent and fast-acting neurotoxin^6^. Clinical evidence has supported the role of excitotoxicity mediated by NMDAR, an ionotropic Glu receptor, in traumatic brain injury^7,8^ and cerebral ischemia^9,10^, both of which are acute CNS insults accompanied by cognitive deficits^11,12^. Furthermore, females are more frequently reported as patients with post-concussive symptoms, which are a combination of symptoms that occur after traumatic brain injury and include difficulty in concentrating and memory loss, in the existing literature than males^13^. Similarly, women experience more severe cerebral ischemic strokes and worse post-stroke cognitive impairments^14,15^.

In healthy populations, sex differences in cognitive function have been well studied^16–18^, but they are not stable and undergo dynamic change across the lifespan. According to Gur and Gur^19^, there was no correlation between age and any of the cognitive measures in women. Men, on the other hand, lost attention, verbal memory, spatial memory, and spatial abilities as they aged. Comparatively, in a longitudinal study of marmosets, cognitive impairment occurred earlier in females than in males, and it seems to be more prevalent for discrimination than in reversal learning^20^.

Our understanding of how sex-related neural information transmission evolves with age is still in its infancy. Chronic excitotoxicity has been related to Alzheimer’s disease^21,22^, which typically onsets in old age and is accompanied by cognitive deficits^23^, and has a different prevalence in men and women^24^. Recent research using mouse models revealed that male and female rodents had distinct excitatory glutamatergic synaptic inputs in both striatal brain regions and the hippocampus^25,26^, because estrogen regulates glutamatergic synaptic transmission by binding to different types of estrogen receptors^27^. Treatment with testosterone, on the other hand, enhanced the performance of aged male mice in the Morris water maze test and increased the level of NMDAR1 expression^28^. Furthermore, Glu-induced acute neurotoxicity in healthy individuals might be masked by the efficiency of normal cellular uptake mechanisms in removing Glu from the synaptic cleft^6,29^ and the potential neuroprotective effects of the sex steroids^30,31^. In other words, cognitive sex differences in healthy individuals are likely to be the phenotype of their susceptibility to the fast-acting and transient excitotoxicity induced by Glu because sex hormone regulation in the glutamatergic system changes with age, resulting in age-related cognitive sex differences.

Proteins perform most of the work in living cells, and changes in the expression of neurotransmission-related proteins resulted in age-dependent differences in male and female brain functioning^32,33^. To become functional, such neurotransmission-related proteins rapidly interacted with one another as protein complexes, which are functional units of proteome organization and are responsible for the majority of biological processes, such as biomedical pathways and signaling cascades in the neuron^34,35^. Synaptic transmission, for example, is dependent on transient and stable protein–protein interactions between the hundreds of components that form the pre- and post-synaptic compartments^36,37^. Instead of studying single proteins, researchers can gain a better understanding of the structure and function of the nervous system by studying protein complexes. Currently, clinical research is prone to using protein complexes to predict the mechanisms of neurodegenerative diseases such as Alzheimer’s disease (AD) and to develop novel treatments in response^38–40^.

Protein complexes are not permanent; their dynamic assembly is fundamental to inducing cellular responses to various internal and external stimuli, and the individual protein complexes involved in a signaling pathway assemble in different compartments at different times^41^. As a result, in order to gain a better understanding of how cells reorganize at a system level in the context of age-dependent cognitive sex differences, this study utilized both human behavior assessment (Wisconsin Card Sorting Test (WCST)) and molecular experiment (label-free proteomics analysis) at the protein global level to investigate the molecular mechanisms behind age-related cognitive sex differences in WCST performance in healthy men and women.

## Results

### Demographic data

The subjects were 89 males and 110 females, with a mean age of 45.6 ± 19.3 years (range 20–70 years). Age differed between men and women (BF = 10.7, 95% CI [2.74, 13.2]), and although a sex difference in education level was discovered (BF = 4.9), the evidence was moderate.

In each age group, differences in age and education level between men and women were not adequately supported. However, there were significant differences in education level among the three age groups (see Table 1).

**Table 1:** Demographic data of subjects.

		Male	Female	Education level	Age	95% CI
Young adult		22 (31.4%)	48 (68.6%)			
	Primary	1	0	BF = 0.26		
	Secondary and Tertiary	21	48			
		21.2 ± 1.50	21.1 ± 3.06		BF = 0.2	[-1.40, 1.44]
Middle-aged adult		32 (54.2%)	27 (45.8%)			
	Primary	19	9	BF = 4		
	Secondary and Tertiary	13	18			
		53.2 ± 6.36	49.4 ± 7.93		BF = 1.42	[0.05, 7.33]
Elderly		35 (50%)	35 (50%)			
	Primary	30	33	BF = 0.81		
	Secondary and Tertiary	5	2			
		65.0 ± 3.59	65.2 ± 3.01		BF = 0.18	[-1.55, 1.58]
Inter-groups				BF > 100		

Data was presented as mean ± SD by general linear model.

BF, Bayes factor; 95% CI, 95% credible interval of difference male against female; Primary, primary education; Secondary and Tertiary, secondary and tertiary education.

### Sex differences in cognitive performance

**Figure 1:**
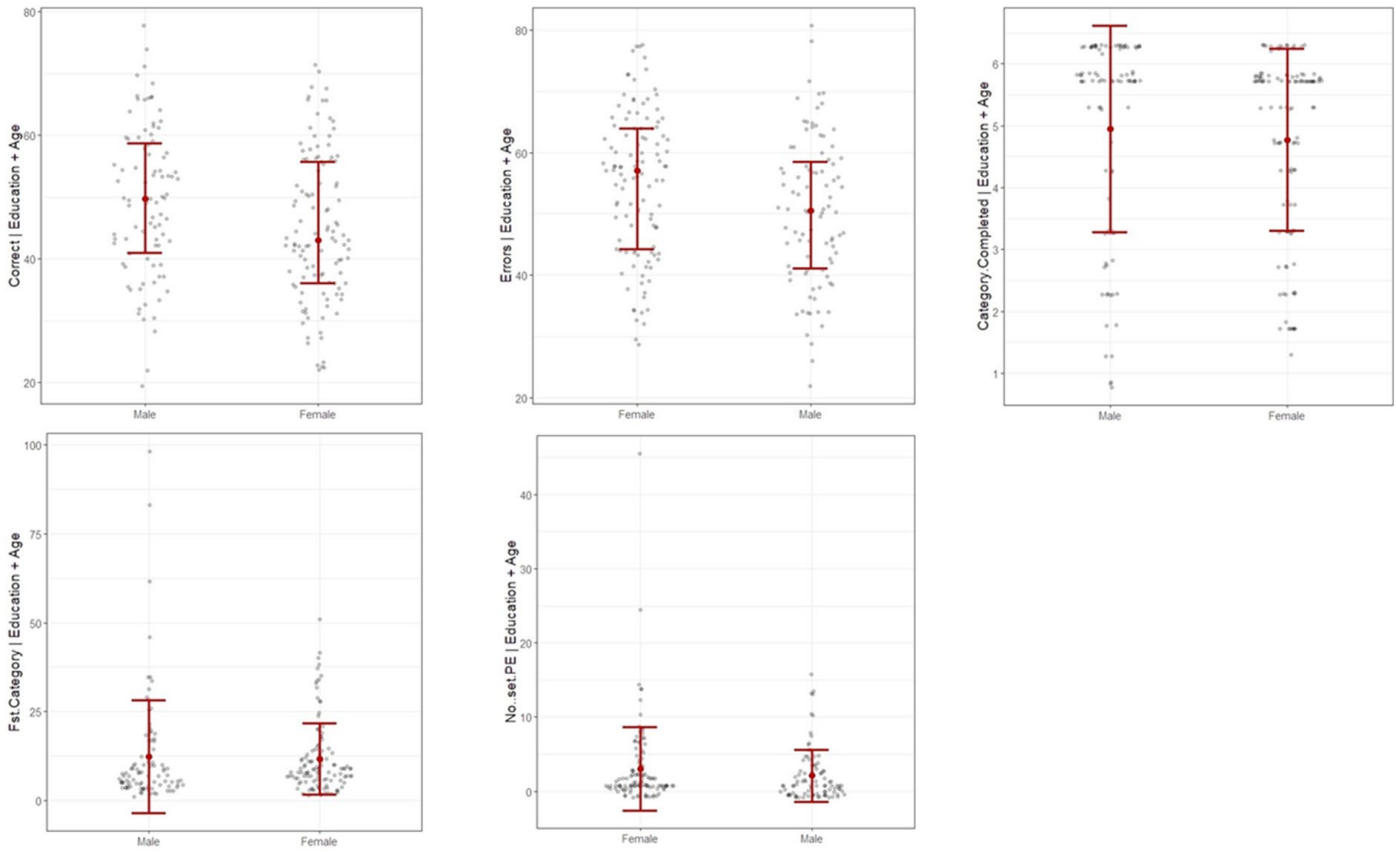
Sex differences in WCST sub-scores after controlling age and education level. Graphs were generated using *flexplot* package in R-programming ver. 4.1.2. Correct = %Corrects, Errors = %Errors, Fst Category = 1st Category, No..set PE = PE.

As shown in Fig. 1, males performed better in %Corrects and had fewer %Errors, with strong evidence to reject the null hypothesis. Weak evidence suggests the presence of sex differences in the other three scores: Category Completed, 1st Category, and PE. Table 2 shows the parameter estimation of sex disparities in those WCST sub-scores.

**Table 2:** The parameter estimation of sex differences in WCST sub-scores.

	Male	Female	BF	95% CI	Cohen’s d
%Corrects	48.8 ± 12.3	45.5 ± 12.6	193	[1.40, 8.23]	0.39
%Errors	51.1 ± 12.2	54.5 ± 12.6	193	[− 8.31, − 1.30]	0.39
Category completed	4.89 ± 1.68	4.81 ± 1.51	1.54	[− 0.27, 0.63]	0.12
PE	2.25 ± 3.55	2.94 ± 5.68	2.48	[− 2.32, 0.38]	0.20
1st Category	12.8 ± 16.1	11.3 ± 10.1	1.86	[− 3.05, 4.31]	0.05

Data was presented as mean ± SD by general linear model.

BF, Bayes factor; 95% CI, 95% credible interval of difference male against female.

### Changes in sex differences in WCST performance with age

**Figure 2:**
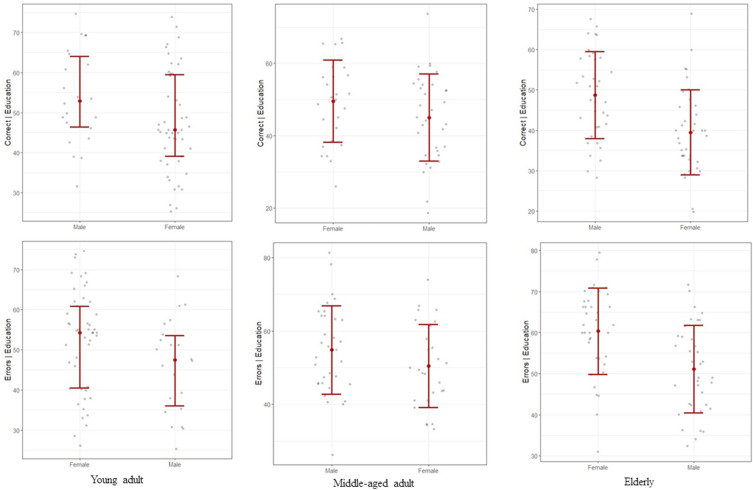
Change of sex differences in WCST scores %Corrects and %Errors with age. Graphs were generated using *flexplot* package in R-programming ver. 4.1.2. Correct = %Corrects, Errors = %Errors.

Age-related sex differences were found in the same two WCST scores: %Corrects and %Errors. As shown in Fig. 2, males scored better in %Corrects (BF = 7.84, 95% CI [− 0.35, 12.6]) and had fewer total errors (BF = 7.58, 95% CI [− 12.7, 0.2]) in the young adult group; however, this sex difference reversed in the middle-aged adult group (%Corrects: BF = 2.09, 95% CI [− 1.27, 11.1]; %Errors: BF = 2.08, 95% CI [− 11.0, 1.54]). Nevertheless, there was insufficient evidence to support sex differences in these two scores in both young and middle-aged adult groups. While male dominance in %Corrects (BF > 100, 95% CI [4.32, 14.6], d = 0.89) and %Errors (BF > 100, 95% CI [− 14.6, − 4.29], d = 0.90) have been found in the elderly group, as in the young adult group, with decisive evidence to support it. In all three age groups, there was weak evidence to support the alternative hypothesis for the other three WCST scores: Category complete, 1st Category and PE (BF < 4).

### Identification and relative quantification of differentially expressed proteins between males and females

**Figure 3:**
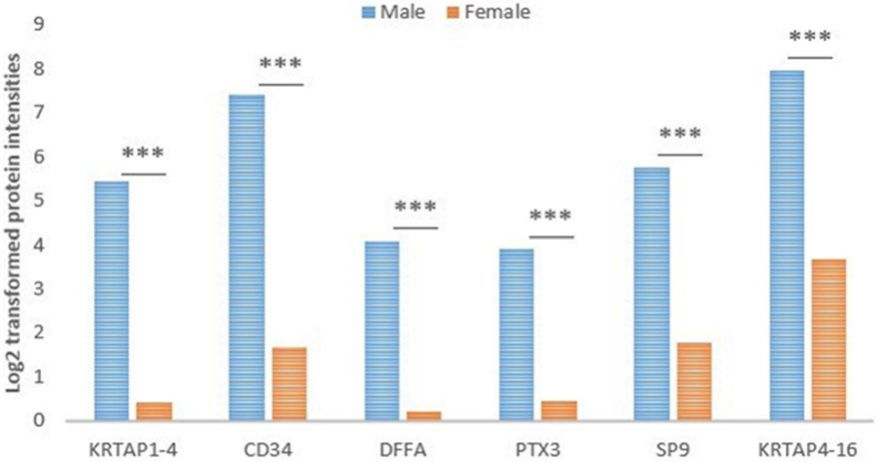
Protein intensities comparison of those 6 DEPs upregulated in males. ***FDR ≤ 0.01.

The label-free proteomics analysis detected 19,640 proteins in each sample after applying protein FDR = 0.1. We discovered 52 proteins that were differentially expressed between male and female research subjects (FDR < 0.01) (see Table 3), with only 6 of those 52 differentially expressed proteins (DEPs) being upregulated in males (see Fig. 3).

**Table 3:** Differentially expressed proteins between males and females detected by LIMMA, FDR = 1%.

Number	Protein IDs	Gene name	Protein name	Fold change (Male/Female)	FDR
1	A6NGW2	STRCP1	Putative stereocilin-like protein	− 9.87378	1.2004E−12
2	P28068	HLA-DMB	HLA class II histocompatibility antigen, DM beta chain	− 10.0593	1.9025E−07
3	P0C5Y4	KRTAP1-4	Keratin-associated protein 1–4	5.006562	5.166E−06
4	O00635	TRIM38	E3 ubiquitin-protein ligase TRIM38	− 7.38556	9.0655E−06
5	P04731	MT1A	Metallothionein-1A	− 5.80523	2.2525E−05
6	P32297	CHRNA3	Neuronal acetylcholine receptor subunit alpha-3	− 5.72918	2.3905E−05
7	C9JL84	HHLA1	HERV-H LTR-associating protein 1	− 5.64453	2.7304E−05
8	O75084	FZD7	Frizzled-7	− 5.46865	2.7304E−05
9	O95221	OR5F1	Olfactory receptor 5F1	− 4.76125	3.4929E−05
10	P16298	PPP3CB	Serine/threonine-protein phosphatase 2B catalytic subunit beta isoform	− 4.81837	3.4929E−05
11	A0A1B0GV85	REELD1	Reelin domain-containing protein 1	− 4.93878	3.4929E−05
12	B4E2M5	ANKRD66	Ankyrin repeat domain-containing protein 66	− 6.80293	8.8842E−05
13	P25311	AZGP1	Zinc-alpha-2-glycoprotein	− 6.08555	0.00013846
14	P08620	FGF4	Fibroblast growth factor 4	− 5.4998	0.00013846
15	P28906	CD34	Hematopoietic progenitor cell antigen CD34	5.718257	0.00016083
16	P02749	APOH	Beta-2-glycoprotein 1	− 5.84757	0.00016748
17	P0C0L4	C4A	Complement C4-A	− 1.67486	0.00016748
18	O00273	DFFA	DNA fragmentation factor subunit alpha	3.878624	0.0003333
19	P38117	ETFB	Electron transfer flavoprotein subunit beta	− 3.96004	0.00036524
20	P14061	HSD17B1	17-beta-hydroxysteroid dehydrogenase type 1	− 4.51463	0.00050686
21	P01024	C3	Complement C3	− 1.22015	0.00057158
22	P04275	VWF	von Willebrand factor	− 3.89944	0.00060659
23	P04004	VTN	Vitronectin	− 4.00878	0.0006814
24	P02766	TTR	Transthyretin	− 4.64038	0.0006814
25	P20338	RAB4A	Ras-related protein Rab-4A	− 5.6758	0.0006814
26	P05155	SERPING1	Plasma protease C1 inhibitor	− 4.44992	0.0006814
27	O43353	RIPK2	Receptor-interacting serine/threonine-protein kinase 2	− 4.3834	0.0006814
28	P0DMS9	TMIGD3	Transmembrane domain-containing protein TMIGD3	− 4.81477	0.00069802
29	O75096	LRP4	Low-density lipoprotein receptor-related protein 4	− 3.74001	0.00090466
30	P13861	PRKAR2A	cAMP-dependent protein kinase type II-alpha regulatory subunit	− 4.93194	0.00093903
31	P26022	PTX3	Pentraxin-related protein PTX3	3.44284	0.00158262
32	P0CG40	SP9	Transcription factor Sp9	3.946251	0.00284037
33	O15397	IPO8	Importin-8	− 4.82519	0.00305309
34	G5E9R7	KRTAP4-16	Putative keratin-associated protein 4–16	4.263715	0.00313433
35	O95674	CDS2	Phosphatidate cytidylyltransferase 2	− 4.39524	0.00343441
36	O75881	CYP7B1	Cytochrome P450 7B1	− 4.64784	0.00349086
37	A0A075B6I0	IGLV8-61	Immunoglobulin lambda variable 8–61	− 2.62925	0.00349086
38	P28331	NDUFS1	NADH-ubiquinone oxidoreductase 75 kDa subunit, mitochondrial	− 4.06335	0.00375275
39	P01042	KNG1	Kininogen-1	− 5.17065	0.00387168
40	O75891	ALDH1L1	Cytosolic 10-formyltetrahydrofolate dehydrogenase	− 3.43624	0.00418803
41	P01008	SERPINC1	Antithrombin-III	− 4.40148	0.00418803
42	P36955	SERPINF1	Pigment epithelium-derived factor	− 4.6215	0.00424301
43	P14652	HOXB2	Homeobox protein Hox-B2	− 2.88034	0.00549476
44	P10412	H1-4	Histone H1.4	− 2.86669	0.00590983
45	O75427	LRCH4	Leucine-rich repeat and calponin homology domain-containing protein 4	− 4.00204	0.00614704
46	P05783	KRT18	Keratin, type I cytoskeletal 18	− 4.18557	0.00756386
47	P08603	CFH	Complement factor H	− 3.11493	0.00756386
48	P04264	KRT1	Keratin, type II cytoskeletal 1	− 1.29099	0.00756386
49	O14980	XPO1	Exportin-1	− 4.08967	0.00849615
50	P0DOX7		Immunoglobulin kappa light chain	−1.35981	0.00863139
51	O15382	BCAT2	Branched-chain-amino-acid aminotransferase, mitochondrial	− 3.24202	0.00881399
52	P22897	MRC1	Macrophage mannose receptor 1	− 3.98199	0.00972859

### Overrepresentation analysis of DEPs

**Figure 4:**
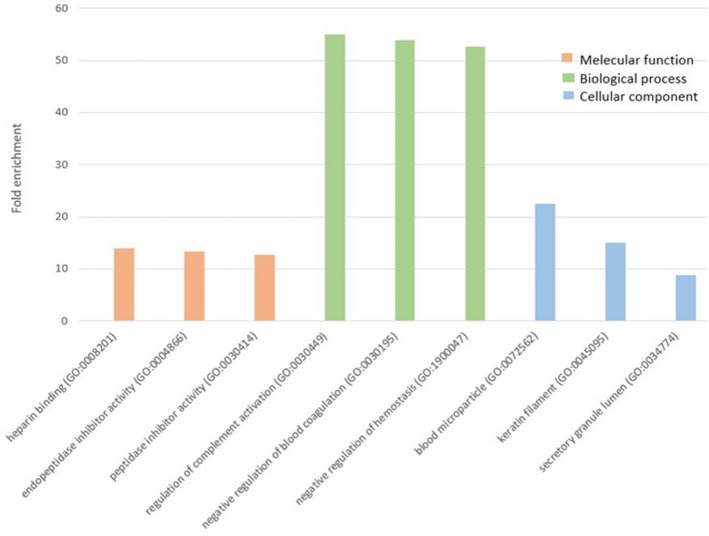
The top three processes in each GO class with DEPs significantly enriched.

To gain insight into the biological changes after activation of the glutamatergic system between both sexes. PANTHER overrepresentation analysis was employed to determine if such DEPs were enriched in certain groups based on the following three Gene annotation (GO) classes: molecular function (MF), biological process (BP), and cellular component (CC). The results showed that in the MF class, the DEPs involved in heparin binding (GO:0008201) were the most significantly enriched (Fold enrichment = 14, FDR = 0.025), and in the BP class, the DEPs involved in the regulation of complement activation (GO:0030449) were the most significantly overrepresented (Fold enrichment = 55.1, FDR = 0.016), while in the CC class, the DEPs involved in blood microparticle (GO:0072562) were the most significantly enriched (Fold enrichment = 22.4, FDR = 7.14E-6). See Fig. 4.

### Pathway enrichment analysis

The analysis of the KEGG pathways^42–44^ indicated that two significantly enriched pathways were found: complementing and coagulation cascades (has:04610) and Staphylococcus aureus infection (has:05150), see Table 4.

**Table 4:** KEGG pathways the differentially expressed proteins enriched.

Number	Pathway ID	Pathway	Mapped DEPs	Fold enrichment	FDR
1	hsa04610	Complement and coagulation cascades	VTN, C4A, KNG1, CFH, SERPINC1, SERPING1, VWF, C3	23.1	3.62E−6
2	hsa05150	Staphylococcus aureus infection	C4A, RING7, CFH, KRT18, C3	12.8	0.032

### Protein–protein interaction analysis of DEPs

**Figure 5:**
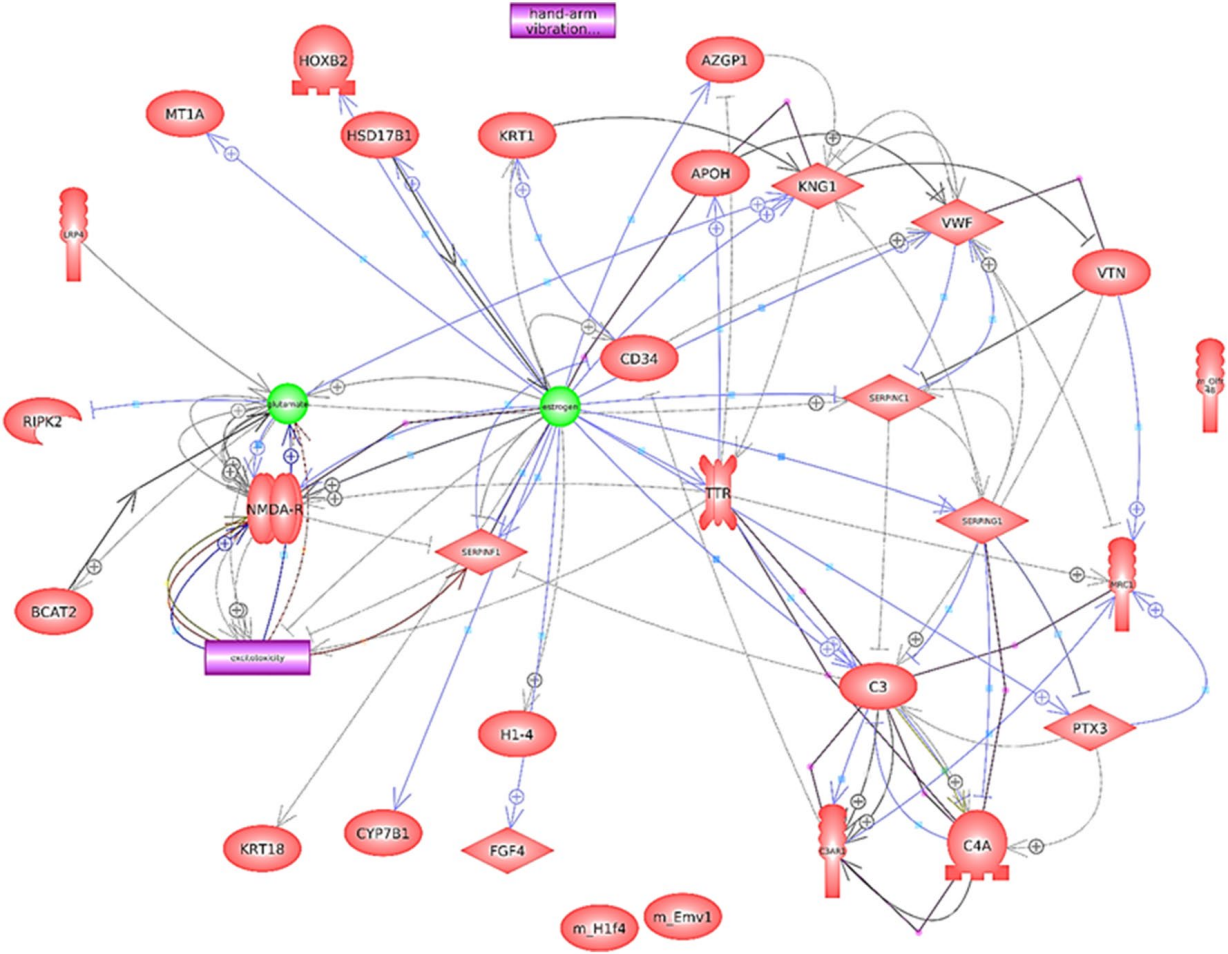
Protein–protein interaction networks of 26 DEPs. The purple line indicates binding, the blue line indicates expression, the black line indicates chemical reactor, the solid gray indicates molecular transport, and the gray dotted line indicates regulation. The plus sign indicates positive regulation.

**Figure 6:**
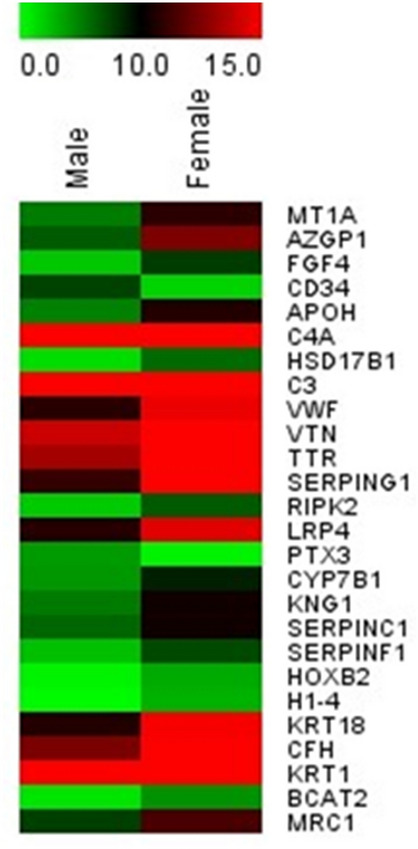
Expression levels of such 26 DEPs between males and females. The expression gradient is from green (minimum) to red (maximum).

As illustrated in Fig. 5, 26 of the 52 DEPs interacted with one another, including protein complement 3 (C3), which is the central component of the complement system and is directly linked to the NMDA receptors (NMDARs). The data also suggested that the excitotoxicity induced by Glu could be mediated via NMDARs activity and estrogen-regulated NMDAR function. The majority of these 26 DEPs were shown to be upregulated in females (see Fig. 6).

### Sex-dependent protein complexes analysis

To investigate the dynamic assembly of protein complexes stimulated by the WCST test, 19,640 previously identified proteins were submitted to the COMPLEAT database.

**Figure 7:**
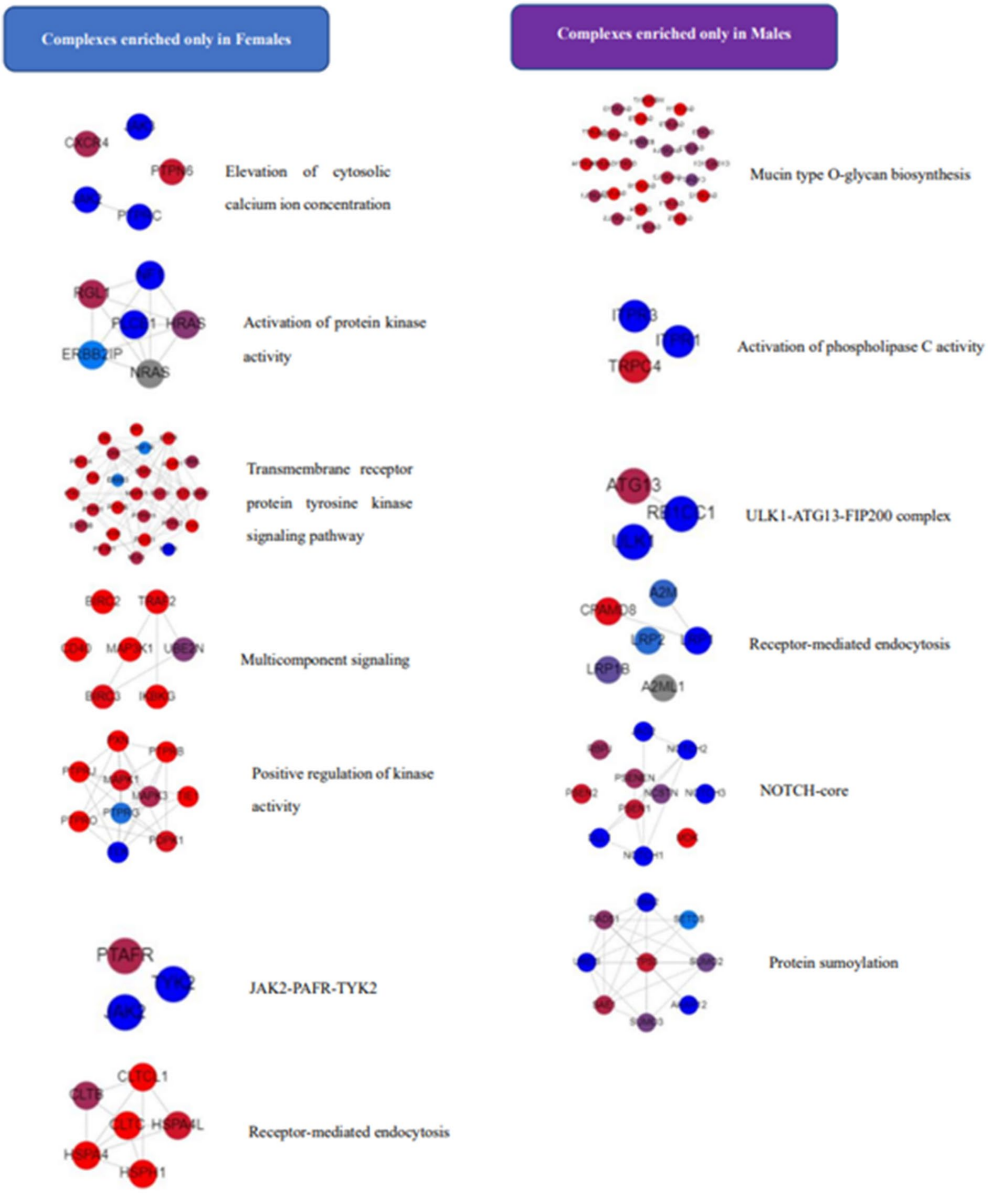
Neurotransmission-related protein complexes are only enriched in males or females. Graph was generated on the website of COMPLEAT. Color gradient varies from red (max input score) to blue (min input score). Dashed lines indicate interactions.

We found seven neurotransmission-related protein complexes selectively enriched in female samples: the elevation of cytosolic calcium ion concentration complex (*P* = 4.45E−04), activation of protein kinase activity complex (*P* = 6.99E−04), transmembrane receptor protein tyrosine kinase signaling pathway complex (4.38E−03), Multicomponent signaling complex (6.90E−03), positive regulation of kinase activity complex (*P* = 7.63E−03), JAK2-PAFR-TYK2 complex (7.82E−03), and receptor-mediated endocytosis complex (9.41E−03). In males, mucin type O-glycan biosynthesis complex (*P* = 6.52E−03), activation of phospholipase C activity complex (*P* = 3.98E−03), ULK1-ATG13-FIP200 complex (*P* = 1.31E−03), receptor-mediated endocytosis (*P* = 8.68E−03), NOTCH-core complex (*P* = 9.29E−03), and protein sumoylation complex (*P* = 9.06E−03) were selectively present, see Fig. 7.

### The dynamic assembly of sex-dependent protein complexes with age

**Figure 8:**
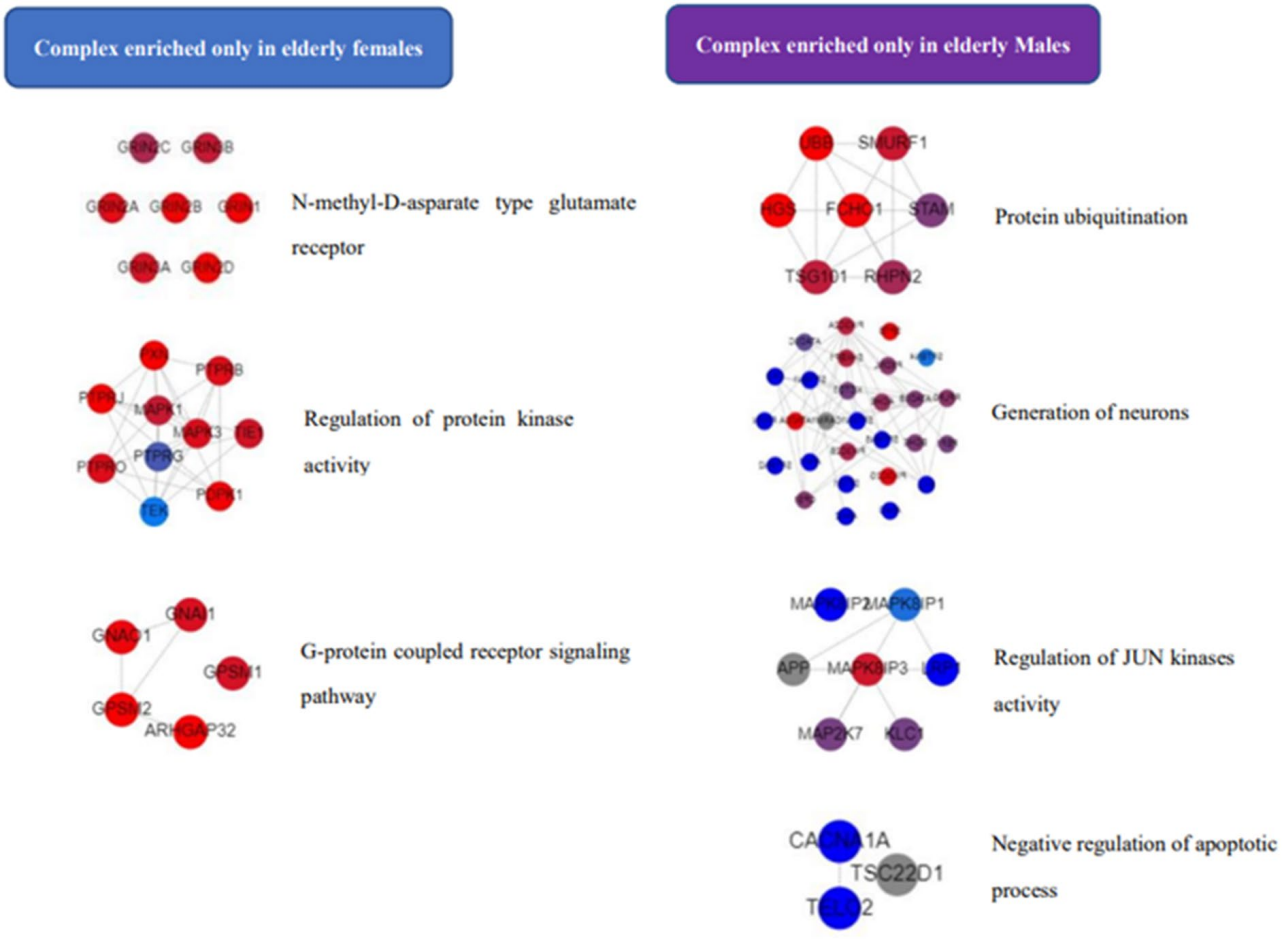
Neurotransmission-related protein complexes are enriched only in elderly males or females. Graph was generated on the website of COMPLEAT. Color gradient varies from red (max input score) to blue (min input score). Dashed lines indicate interactions.

As shown in Fig. 8, we detected that the N-methyl-D-aspartate type glutamate receptor (NMDAR) complex (*P* = 0.005), regulation of protein kinase activity complex (*P* = 0.009), and G-protein coupled receptor signaling pathway complex (*P* = 0.008) were solely concentrated in elderly females. There were four neurotransmission-related complexes that were only enriched in elderly men: protein ubiquitination complex (*P* = 0.004), generation of neurons complex (*P* = 0.002), regulation of JUN kinases activity complex (*P* = 0.008), and negative regulation of apoptotic process complex (*P* = 0.007). Furthermore, the elevation of cytosolic calcium ion concentration complex (*P* < 0.005) was only found in females across all three age groups.

The regulation of neuron differentiation complex (*P* = 0.003), NOTCH-Core complex (*P* = 0.0007), and activation of protein kinase activity complex (*P* = 0.0009) were found to be enriched only in females in the elderly samples; however, these complexes existed as common complexes in the middle-aged group and were not seen in young adult samples. While the axon guidance complex was present as a common complex in the young adult group, it was concentrated only in females in both middle-aged (*P* = 0.0007) and elderly (*P* = 0.002) groups. Furthermore, the nervous system development complex was found to be enriched only in males in both middle-aged (*P* = 0.009) and elderly (*P* = 0.008) groups, but not in young adults. The histone H3-K4 methylation complex was not present in the young adult group, was common in the middle-aged group, and was enriched only in males in the elderly group (*P* = 0.007).

## Discussion

In the current study, to gain a better understanding of how cognitive sex differences evolve with age in healthy Thai subjects, frontal cortical cognitive performance was assessed using WCST. Males outperformed females in two of the five WCST sub-scores: %Corrects and %Errors, with a higher percentage of total corrects and a lower total error rate. We also found that the sex difference in these two WCST sub-scores changed with aging, and this difference becomes noticeable in those over 60. This is congruent with the findings of a study by Whitley, et al.^45^, which demonstrated that in UK residents, men and women subtraction scores and numerical problem-solving ability show different trends with aging beginning at 60.

The WCST is a test of cognitive flexibility, that is, the ability to adjust behavioral response mode in the face of changing conditions^46,47^, it is commonly used to test the function of the frontal cortex, particularly the prefrontal cortex^48^. Its sub-scores %Corrects and %Errors show specific domains of cognitive function, which correspond to the activity of certain brain regions. For example, %Corrects reflects conceptualization and attention, and a previous study showed that multiple areas of the prefrontal lobe may participate in information processing during an attentional shift in the WCST^49^. While %Errors reflecting non-specific cognitive impairment and lesions in the frontal and temporal lobes have been linked to a large number of WCST total errors^50,51^. This score has also been demonstrated to be adversely connected with the Full-Scale Intelligence Quotient (FSIQ)^52,53^, a measure of a person’s overall level of general cognitive and intellectual functioning^54^. Despite the standardization, validity, and reliability of the WCST as a stand-alone cognitive evaluation in healthy individuals of different ages and education levels have been established^55^, a battery of cognitive assessments is required to unveil the sex difference in different cognitive domains.

At the molecular level, the DEPs between sexes were most significantly enriched in complement cascade, which is a major component of the innate immune system^56^. One of the fundamental functions of complement activation in the healthy brain is to protect neurons from potentially harmful toxic stimuli^57^. In this study, data from protein–protein interaction analysis indicated that complement component 3 (C3), a central component of the complement system, was connected to NMDAR-mediated Glu-induced excitotoxicity. This conclusion is consistent with the previous finding that C3 was discovered to be expressed in the rat hippocampus following acute excitotoxic injury^58^. This result also demonstrated that estrogen, which may have a neuroprotective effect^30,31,59^, controls NMDAR function directly. Moreover, complement system components such as C3 and C4A, as well as regulators such as CFH, VTN, and SERPING 1, were detected in both males and females. Considering the blood drawn from the subjects after the WCST test, the complement activation is likely associated with the generation of potential excitotoxicity induced by Glu via NMDAR. The majority of the DEPs were upregulated in female subjects when compared to males, suggesting that females may be more susceptible to such excitotoxicity. as evidenced by their performance in score %Errors, which reflect nonspecific cognitive impairment and negatively connected with FSIQ.

To be functional, such DEPs are dynamically assembled as protein complexes, and this study uncovered a number of sex-biased protein complexes. For example, the elevation of the cytosolic calcium ion concentration complex was only found in the female samples. In classic excitotoxicity, impaired Glu transporter function leads to increased extracellular Glu, which elicits a massive influx of calcium into neurons via NMDARs^60^, and elevated calcium ions ultimately contribute to irreversible excitotoxic injury^61^. Protein kinase C (PKC), a family of protein kinase enzymes, has been shown to regulate NMDARs trafficking and gating^62^, and upregulating cellular PKC activity can exacerbate neurotoxicity mediated by NMDA receptor activation^63^. In addition, the platelet-activating factor receptor (PAFR) interacts with Tyk2 to promote Janus kinase 2 (Jak2) activation^64^. Jak is a type of protein tyrosine kinase^65^, and data from experimental mice and clinical observations have revealed multiple signaling events mediated by Jak in innate and adaptive immunity^66^.

Besides that, the activation of the phospholipase C (PLC) activity complex was identified only in males. PLC activation is associated with enhanced NMDAR function^67^, whereas PLC inhibition suppresses NMDAR-dependent long-term depression^68^. Another complex that is only found in men is the ULK1-ATG13-FIP200 complex, which mediates mTOR signaling^69,70^. A previous study has demonstrated that NMDAR activation regulates sociability through its effects on the mTOR signaling pathway^71^. Radiske et al.^72^ revealed that hippocampal NMDARs drive local protein synthesis via mTOR signaling and may control active memory maintenance, and that the enrichment of the ULK1-ATG13-FIP200 complex in males may offer a compensating effect on excitotoxicity-induced cell injury. Furthermore, protein sumoylation, as a post-translational modification, is essential in various biological processes, and an earlier study has shown that global sumoylation levels shape the immune responses^73^. This is consistent with the findings of the individual DEPs analysis and adds to its support. Additionally, previous research shows that one function of multi-component signaling is to mediate environmental stress^74,75^, and its selective enrichment in females suggests that the aforementioned sex-specific neurotransmission-related protein complexes are likely to be assembled transiently in response to the WCST stimulation. However, the DEPs were extracted from subjects’ blood rather than brain tissues; previous study indicated that plasma proteins permeate the healthy brain^76^, and that the majority of neurotransmission-related proteins are synthesized locally^77,78^. The data in the current study provides preliminary evidence that implies that differences in performances in WCST scores %Corrects and %Errors between male and female subjects might be due to their different susceptibilities to the potential NMDAR-mediated ecotoxicity.

Protein complexes are not permanent; their dynamic assembly is fundamental to inducing cellular responses to various internal and external stimuli, and the individual protein complexes involved in a signaling pathway assemble in different compartments at different times^41^. The N-methyl-D-aspartate type glutamate receptors (NMDARs) complex and its regulators tyrosine kinase (TK)^79,80^ and mitogen-activated protein kinase (MAPK)^81^, and G-protein coupled receptors^82,83^ were exclusively enriched in elderly females. While in elderly males, regulation of JUN kinase activity and protein ubiquitination complexes were only found in them. JUN kinase has been reported to mediate glutamate-induced excitotoxicity^84^, with JUN kinase controlling NMDAR-evoked presynaptic glutamate release as a possible mechanism^85^, and NMDAR activation leads to related protein ubiquitination^86^. This finding implied that subjects in the elderly group may have experienced higher levels of potential excitotoxicity mediated by NMDAR than the other two age groups. This is consistent with the hyperfunction theory of aging^87^, which states that aging is not functional decline but is caused by cellular hyperfunction—a function that was not switched off upon its completion—that results in age-related diseases. This theory linked aged-related functional loss to inappropriate activation of signaling pathways. Thus, later in life, although both elderly male and female subjects may experience higher-than-optimal levels of NMDAR functions, elderly females lose the neuroprotective effects of estrogen after menopause^88,89^, resulting in the loss of brain environment homeostasis and impaired cognitive function, as evidenced by their cognitive performance. This provided a possible explanation for why the sex differences in %Corrects and %Errors were only significant in the elderly group, and NMDAR protein complex enrichment in serum could be suggested as a potential indication for predicting cognitive aging in healthy Thai females.

There are several limitations to our study. First, in an attempt to obtain a sample that is approximately representative of the population, the sample covers a range of subjects with varied ages, education levels, socioeconomic statuses, and occupations; these also increase the variability of the sample. Second, except for age and education level, other confounders such as socioeconomic status and occupations were not controlled for in this study, despite the fact that both potentially contribute to the sex difference in cognitive performance. Third, only the subject’s education level was obtained; the number of years of study was not collected, and further research needs to better assess the contribution of years of education. Fourth, while some neurochemicals do cross the blood-brain barrier^90^, the majority of them might be locally synthesized in the brain^91,92^, and future studies directly comparing protein expression profiles in the brain tissues between males and females are needed to replicate our finding. Last, owing to the moderate sample size, generalizing the findings of this study should be done with caution.

## Conclusion

The current study showed a preliminary connection between sex differences in cognitive performance in WCST in healthy Thai male and female subjects and their different susceptibilities to the potential NMDAR-mediated excitotoxicity that is modulated by sex steroids. Because of the efficiency with which molecular uptake mechanisms remove Glu from the synaptic cleft and neuroprotective effects of sex steroids, the acute and transient excitotoxicity in healthy populations might be masked; consequently, the WCST sub-score %Errors could be an indicator of potentially excitotoxic levels in them. Additionally, NMDAR protein complex enrichment in serum could be suggested as a potential indication for predicting cognitive aging in healthy Thai females. To our best knowledge, this is the first study to investigate the association between susceptibility to excitotoxicity and age-dependent cognitive sex differences in healthy populations, and our findings contribute to a better understanding of the neural mechanisms underlying the change of sex differences in cognitive functions with aging assessed by WCST.

## Materials and methods

### Study subjects

One hundred and ninety-nine healthy subjects aged 20–70 years were recruited, as described in a previous study from our lab^93^, and they were assigned into three age groups: (1) the young adult group, age range from 20 to 34 years (n = 70), (2) the middle-aged adult group, age range from 35 to 59 years (n = 59), and (3) the elderly group, age range from 60 years and above (n = 70). All subjects were of Thai ethnicity, to reduce the possibility of confounding by population stratification^94^. The research was approved by the Institutional Review Board (IRB) of Naresuan University, Thailand (COA No. 0262/2022). All methods were performed in accordance with relevant guidelines and regulations (Declaration of Helsinki). Participation was voluntary, and a written consent form was obtained from each participant involved.

### Cognitive assessment

The cognitive performance of each research subject was assessed by the Wisconsin Card Sorting Test, specifically, five WCST sub-scores were used in this study: the percentage of total corrects (%Corrects), the percentage of total errors (%Errors), the number of categories completed (Category completed), the perseverative errors (PE), and trails to complete the first category (1st Category), as mentioned by a prior study^93^.

### Blood sample collection

A 3 mL cubital vein blood sample was collected from each enrolled subject immediately after completing the WCST test, allowing us to explore the dynamic assembly of protein complexes in males and females from different age groups. Blood is an ideal biological sample for this study because it contains proteins from numerous cells and tissues and is convenient to assess^95^. The blood sample was centrifuged at 3000 rpm for 5 min. The serum was then transferred into a 1.5-ml microcentrifuge tube and stored at – 80 °C in a refrigerator for future use. All samples were coded to ensure anonymity.

### Label-free quantitative proteomics analysis

As described by our previous study^96^, the label-free shotgun proteomics processes were performed by the Functional Proteomics Technology Laboratory, National Centre for Genetic Engineering and Biotechnology, Pathum Thani, Thailand, including protein preparation, peptide digestion, Liquid Chromatography with tandem mass spectrometry (LC–MS/MS) analysis, protein identification, and protein quantitation.

Briefly, five micrograms of protein samples were induced with 10 mM dithiothreitol, alkylated with 30 mM iodoacetamide, digested with sequencing grade porcine trypsin (1:20 ratio) for 16 h at 37 °C. The prepared tryptic peptide sample of each subject was injected individually into an Ultimate3000 Nano/Capillary LC System (Thermo Scientific, UK) coupled to a Hybrid quadrupole Q-Tof impact II™ (Bruker Daltonics) equipped with a Nano-captive spray ion source. Mass spectra (MS) and MS/MS spectra were obtained in the positive-ion mode at 2 Hz over the range (m/z) 150–2200. To minimize the effect of experimental variation, three independent MS/MS runs were performed for each sample.

MaxQuant 1.6.6.0 was used to quantify and identify the proteins in the individual samples using the Andromeda search engine to correlate MS/MS spectra to the Uniprot *Homo sapiens* database^97^. The proteins were identified by a 10% protein false discovery rate (FDR), carbamidomethylation of cysteine as fixed modification, and the oxidation of methionine and acetylation of the protein N-terminus as variable modifications. Only proteins with at least two peptides and at least one unique peptide were considered to be identified and used for further data analysis.

### Bioinformatic analysis

Before any analysis, data cleansing and preprocessing were performed using Perseus 1.6.15.0^98^. Differentially expressed proteins (DEPs) between both sexes were detected by the Linear Model for Microarray Data (LIMMA) approach within R-programming ver. 4.1.2^99^, with FDR set at 1%.

PANTHER database analysis tools (ver. 16) were employed for the overrepresentation analysis of DEPs^100^. Pathway analysis was performed using the Database for Annotation, Visualization, and Integrated Discovery (DAVID) database^101,102^. The protein–protein interaction network was analyzed and visualized by Pathway Studio ver. 12.5^103^. The Complex Enrichment Analysis Tool (COMPLEAT), a web-based data mining and visualization tool for complex-based analysis of high-throughput data sets, as well as analysis and integration of heterogeneous proteomics and gene expression data sets^104^, was employed to investigate the dynamic assembly of protein complexes following the WCST in men and women of different ages. Expression levels of DEPs was visualized using Multi-Experiment Viewer software (MeV, ver.4.9.0)^105^. Benjamini corrected P-values less than 0.05 were considered significant.

### Statistical analysis

Using R-programming ver. 4.1.2, a General Linear Model (GLM) approach paired with Bayesian statistics was applied to analyze sex differences in WCST sub-scores (covarying for age and educational level) and the difference of WCST scores between males and females from each age group (covarying for educational level)^106^. A Bayes factor ≥ 10 indicated a high likelihood of supporting the alternative hypothesis^107^.

The fold changes (FC) of DEPs were calculated and displayed as log_2_^(FC)^.

## Data Availability

The datasets generated during and/or analyzed during the current study are available from the corresponding author on reasonable request.
